# Validity of the multidimensional assessment profile of disruptive behavior in autistic toddlers

**DOI:** 10.1002/jcv2.12233

**Published:** 2024-04-15

**Authors:** Hannah Fipp‐Rosenfield, Jeffrey Grauzer, Megan Y. Roberts, Aaron J. Kaat

**Affiliations:** ^1^ Department of Communication Sciences and Disorders Northwestern University Roxelyn and Richard Pepper Evanston Illinois USA; ^2^ Department of Medical Social Sciences Northwestern University Feinberg School of Medicine Chicago Illinois USA

**Keywords:** autism spectrum disorders, disruptive behavior, rating scales

## Abstract

**Objective:**

Early measurement of atypical disruptive behavior within autistic children is critical for later referrals to behavioral screenings, diagnoses, and services. Disruptive behavior in autistic toddlers is often measured using a categorical approach and identifies the presence or absence of behavior. In contrast, dimensional approaches evaluate behavior on a spectrum of typical to atypical by measuring the clinical salience of disruptive behavior. We sought to assess the validity of the Infant/Toddler version of the multidimensional assessment profile of disruptive behavior (MAP‐DB‐IT), a dimensional approach measurement tool, in a sample of autistic toddlers.

**Methods:**

Autistic toddlers (*n* = 82, *M*_age_ = 33.2 months, SD = 6.28 months) and their mothers received 8 weeks of caregiver‐mediated social communication intervention. Mothers completed the MAP‐DB‐IT and the Infant Toddler Social Emotional Assessment (ITSEA) across three timepoints: before intervention, immediately after intervention, and at 3 months post‐intervention follow‐up. The MAP‐DB‐IT provided scores for three subdomains: temper loss, noncompliance, and aggression (generically or specifically with siblings). Ratings on the MAP‐DB‐IT were compared to the ITSEA using several analytic strategies such as evaluating (a) the internal consistency of the MAP‐DB‐IT domain scores; (b) the convergent validity between the two measures; and (c) its convergent change due to intervention and if this varied by child characteristics.

**Results:**

The MAP‐DB‐IT demonstrated excellent internal consistency across all four subdomains. We evaluated convergent validity and found positive correlations between the (a) ITSEA externalizing and MAP‐DB‐IT aggression domain, (b) ITSEA externalizing and MAP‐DB‐IT aggression with siblings domain, and (c) ITSEA dysregulation and MAP‐DB‐IT temper loss domain.

**Conclusion:**

The MAP‐DB‐IT is a valid measurement tool for disruptive behavior in autistic toddlers. Clinicians should consider the use of the MAP‐DB‐IT for young autistic clients presenting with disruptive behavior to (a) discriminate between early developmentally appropriate tantrums from clinically salient dysregulation, and (b) refer to additional behavioral evaluations and services.

**Key points**

- Disruptive behavior in autistic toddlers is typically measured using a categorical approach, which identifies the presence or absence of behavior.
- Clinicians should consider the use of a dimensional approach measurement tool which assesses normative from clinically salient disruptive behavior by evaluating the behavior's frequency, quality, and context.
- We evaluated the validity of the Infant/Toddler version of the Multidimensional Assessment Profile of Disruptive Behavior (MAP‐DB‐IT), a dimensional approach measurement tool, in a sample of autistic toddlers.
- The MAP‐DB‐IT demonstrates appropriate validity evidence for disruptive behavior in autistic toddlers, including excellent internal consistency and convergent validity between subdomains of the MAP‐DB‐IT and the Infant Toddler Social Emotional Assessment.

## INTRODUCTION

Autism spectrum disorder is a developmental disability clinically defined by deficits (American Psychiatric Association, 2013) in social communication, social interaction, restricted and repetitive behaviors (American Psychiatric Association, 2013; Dwyer et al., 2022; Singer et al., 2022). However, autistic children also present with high rates of co‐occurring disruptive behavior (e.g., aggression, tantrums, noncompliance) (Kaat & Bearss, 2020; Kaat & Lecavalier, 2013). Specifically, within a clinical sample 50% of autistic children were reported to display disruptive behaviors (Kanne & Mazurek, 2011). Furthermore, a review of the literature found that 30% of autistic children met full diagnostic criteria for a disruptive behavior disorder (Kaat & Lecavalier, 2013). Disruptive behavior is common in toddlerhood, as tantrums and dysregulation may be due to children experiencing changes in autonomy as well as increasing environmental demands (Brownell et al., 2007). The display of disruptive behavior is often an external manifestation of a child's internal perspective (e.g., a child may scream to communicate that they are frustrated) (Liu, 2004). As children develop into school‐age, they gain more sophisticated self‐regulation and communication skills, which reduces the frequency of disruptive behavior used for a child to express their wants and needs (Brownell et al., 2007). However, if disruptive behavior persists from toddlerhood into later childhood it is associated with lower language, communication, and academic skills, and increases the risk for co‐occurring disruptive behavior disorders (e.g., oppositional defiant disorder, conduct disorder [CD]) (Ashburner et al., 2008; Chow & Wehby, 2019; Kaat & Bearss, 2020). Therefore, early evaluation of clinically salient disruptive behavior is critical for timely referral to additional screenings, diagnostic evaluation, behavioral services, and facilitating optimal developmental outcomes (Krogh‐Jespersen et al., 2021). Clinicians, that work with autistic children, should consider the use of assessment tools that adhere to a dimensional approach; that operationalizes a spectrum of typical (i.e., developmentally‐expected) to atypical disruptive behavior, rather than categorical approaches that inform if the disruptive behavior is present or absent. Ultimately, dimensional assessment tools further inform characterization of early disruptive behaviors that may negatively impact later development (Wakschlag et al., 2014). However, few disruptive behavior assessment tools that adhere to a dimensional approach are available and have been validated in young autistic children (Wakschlag et al., 2014). Therefore, the current study aims to validate the psychometric properties of the Multidimensional Assessment of Preschool Disruptive Behavior Infant‐Toddler (MAP‐DB‐IT) (Krogh‐Jespersen et al., 2021; Zhang et al., 2023) version, a behavior rating scale following the dimensional approach for multiple types of disruptive behavior, in a sample of autistic toddlers.

The MAP‐DB‐IT is a parent report questionnaire that is comprised of three subdomains: temper loss, noncompliance, and aggression (Krogh‐Jespersen et al., 2021). Temper loss measures the regulation of frustration (e.g., how often does your child suddenly have a temper tantrum that came out of nowhere), noncompliance evaluates a child's internalization of rules (e.g., how often does your child do something dangerous despite repeated warnings), and aggression assesses the capacity to modulate aggressive tendencies (e.g., how often does your child hit, shove, kick, bite, or pinch to try to get something they wanted) (Krogh‐Jespersen et al., 2021; Wakschlag et al., 2014). Most of the aggression questions are relevant to all children, but a subset of questions relate specifically to aggression towards siblings and thus are only asked to those with siblings. The MAP‐DB‐IT was created based on a dimensional approach (Krogh‐Jespersen et al., 2021; Wakschlag et al., 2014). The dimensional approach assesses normative disruptive behavior within childhood from clinically salient disruptive behavior by evaluating the behavior's frequency, quality, and context (Wakschlag et al., 2014). Within the MAP‐DB‐IT behavior is characterized by behavioral frequency (e.g., how often does your child have a temper tantrum that lasted longer than 5 min), quality (e.g., how often does your child have a temper tantrum that came out of nowhere), and context (e.g., how often does your child have a temper tantrum when you were out in public when him/her). Therefore, the specific items on the MAP‐DB‐IT provide a more nuanced distinction between developmentally appropriate and clinically concerning behaviors. For example, when distinguishing developmentally expected versus unexpected irritability (i.e., temper loss) using the MAP‐DB‐IT, during the transition to toddlerhood found that over 40% of toddlers “have a temper tantrum when s/he is hungry, tired, or sick,” but only 3% “suddenly have a temper tantrum that came out of nowhere” (Krogh‐Jespersen et al., 2021). This suggests that temper loss that is “out of nowhere” is exhibited at a lower rate and may be clinically concerning as compared to similar behavior that may be due to environmental circumstances (e.g., sickness, fatigue, hunger). By using a measure informed by the dimensional approach to evaluate early childhood disruptive behavior, clinicians may be able to monitor clinical patterns, and later refer children for behavioral screenings, diagnostic evaluations, or services.

There are various disruptive behavior assessment tools that have been created for autistic children or validated in autistic samples (e.g., the Child Behavior Checklist [CBCL], the Infant Toddler Social Emotional Assessment [ITSEA], and the Aberrant Behavior Checklist [ABC], etc.) (First et al., 2013; Kaat et al., 2014; Offermans et al., 2022). Many of these measures were created based on attributes of both dimensional and categorical approaches. As such, many use diagnostic criteria to determine frequency and severity of disruptive behavior. However, the individual items within these measures may not capture the quality or the context of the disruptive behaviors that are present (Kaat et al., 2019). When accounting for frequency, quality, and context, as done in the MAP‐DB‐IT, individual subdomain scores encompass a variety of items that quantify the dimensionally of multiple facets of a child's disruptive behavior. While this allows for the MAP‐DB‐IT to provide a more nuanced measure of disruptive behavior, the items were not originally developed for autistic toddlers. As such, the MAP‐DB‐IT needs to be evaluated for its ability to identify specific manifestations of behavioral psychopathology within autistic toddlers. By gathering validity evidence for the MAP‐DB‐IT for autistic toddlers, researchers and clinicians may be able to discriminate between early developmentally appropriate tantrums from problematic dysregulation across groups (i.e., the general population and autistic toddlers).

To date no disruptive behavior assessment tool based on the dimensional approach has been validated within a sample of autistic toddlers. Therefore, the primary purpose of the current study was to evaluate the validity of the MAP‐DB‐IT within autistic toddlers. With appropriate validity evidence, the MAP‐DB‐IT may be used to evaluate autistic Toddlers developmentally appropriate behavior from clinically salient behavior, and evaluate differences in disruptive behavior between toddlers from the general population and autistic toddlers. Specifically, we evaluated several types of validity evidence, including (a) the internal consistency of the MAP‐DB‐IT domain scores; (b) the convergent validity of the domains with a more generic measure of toddler behavior (i.e., the ITSEA); and (c) the responsiveness—operationalized as convergent change—of the MAP‐DB to intervention. Further, we evaluated whether the convergent validity between the MAP‐DB‐IT and ITSEA varied based on child characteristics such as child language level or severity of autism (quantified by the autism diagnostic observation schedule [ADOS‐2]).

## METHODS

### Study design

The current study was a secondary analysis of data from a randomized clinical trial (NCT02632773). Participants were recruited continuously between July 2015 and March 2020. This study included 82 mother‐child dyads (*M*_age_ = 33.2 months, SD = 6.28 months). Previous work with the same sample reported 96 mother‐child dyads (Jones et al., 2022); however, the MAP‐DB‐IT was added as an additional survey after study launch and 14 participants did not complete the survey. Participants were randomized to receive either directive or responsive communication interventions. Mothers in both intervention groups received weekly, 1‐h intervention sessions for 8 weeks. Roberts et al. (2022) provides further information about intervention strategies. This study contained three time points: participants were assessed and completed questionnaires before intervention (i.e., T0), immediately after intervention (i.e., T1), and at 3 months post‐intervention follow‐up (i.e., T2).

### Participants

Enrolled participants were from the greater Chicagoland area and were recruited from pediatricians, autism diagnostic programs, and Part C Early Intervention providers (see Table 1). Children were eligible for the study if they were between 24 and 48 months old, had a diagnosis of autism or mothers had concerns about autism, which were later confirmed by an in‐person autism assessment including the ADOS‐2, and had no additional diagnosis that could impact language development (Lord et al., 2012). The original clinical trial evaluated maternal characteristics (i.e., broader autism phenotype) on intervention strategy use; therefore, caregivers were eligible if they were the biological mother to the participating child, spoke English at least 50% of the time with the participating child, and did not have a diagnosis of: Fragile X syndrome, cerebral palsy, schizophrenia, profound hearing loss, or brain or head injury where she lost consciousness.

**TABLE 1:** Mother and child demographic characteristics.

Child baseline (T0) characteristics	
Age	
Months	33.2 (6.28)
Biological sex	
Male	61 (74%)
Race	
White	44 (54%)
Asian	2 (2%)
Black	11 (13%)
More than one race	21 (26%)
Prefer not to answer	4 (5%)
Ethnicity	
Hispanic	32 (39%)
Not Hispanic or Latino	48 (59%)
Prefer not to answer	2 (2%)
Language level	
ADOS‐2 verbal (single word or multi‐word speech)	40 (49%)
ASD severity	
ADOS‐2 severity level moderate and high	81 (99%)
Non‐verbal cognition	
The Mullen scales of early learning‐visual reception	23.51 (6.04)
Mother baseline (T0) characteristics	
Age	
Years	35.23 (5.07)
Race	
White	49 (60%)
Asian	5 (6%)
Black	13 (16%)
Native Hawaiian or other Pacific Islander	1 (1%)
More than one race	7 (9%)
Prefer not to answer	7 (9%)
Ethnicity	
Hispanic	24 (29%)
Not Hispanic or Latino	56 (68%)
Prefer not to answer	2 (2%)
Education	
Without HS	1 (1%)
HS graduate	10 (12%)
Some college	20 (24%)
College graduate from 4‐year college or more	51 (62%)

Abbreviation: ADOS‐2, autism diagnostic observation schedule.

### Measures

#### MAP‐DB‐IT

The MAP‐DB‐IT version is a parent‐report questionnaire that evaluates disruptive behavior in toddlers and was originally created for toddlers within the general population ages 12–24 months (Krogh‐Jespersen et al., 2021; Zhang et al., 2023). Mothers were asked to complete the MAP‐DB‐IT across all time points (i.e., T0, T1, and T2). The questionnaire uses a dimensional approach by distinguishing developmentally‐expected misbehavior within early childhood from clinically salient disruptive behavior (Moreland & Dumas, 2008) by assessing behavioral frequency, quality, and context (Krogh‐Jespersen et al., 2021; Wakschlag et al., 2014).

The MAP‐DB‐IT consists of three subdomains: temper loss, noncompliance, and aggression. Participants completed additional questions if the study child had a sibling. As such, these items formed a 4th subdomain, hereinafter referred to as aggression with sibling items, that captures aggression with sibling and assesses the study child's capacity to modulate aggressive tendencies towards a sibling (e.g., “In the past month, how often does your child hit his/her brother or sister at the slightest provocations”). Items within the temper loss domain (30 items) range from developmentally expected expressions (i.e., tantrums due to frustration) to intense expressions (i.e., dysregulated tantrums that “appear out of the blue”). Items within the noncompliance domain (16 items) range from normative refusal to refusal due to uncooperative disobedience. Items within the aggression domain (20 items) include normal reactive aggression to developmentally unexpected display of intentional or hostile aggression. Items within aggression with sibling items were only provided to participants with siblings (*n* = 43, completed across any timepoint) and captured similar aggression items to the aggression subdomain but as they pertain to interactions with a sibling (five additional items). Within each of the subdomains, parent's reported behavior on a six‐point frequency scale (0 = Never; 1 = Rarely [less than once per week]; 2 = Some [1–3] days of the week; 3 = Most [4–6] days of the week; 4 = Every day of the week; 5 = Many times each day).

For the current study, the MAP‐DB‐IT was scored based on a mean scoring method. If the parent answered more than half the items within a domain, then the items answered were averaged and multiplied by the total number of items within the domain. If the parent answered less than half of the items within the domain, then the domain went unscored (i.e., the subdomain score was also missing). All parents across all time points met the minimum scoring criteria and no domains went unscored (see Table 2).

**TABLE 2:** Child disruptive behavior distributions.

	T0	T1	T2
Completed both MAP‐DB‐IT and ITSEA	82	70	61
MAP‐DB‐IT temper loss	33.67 (23.14)	30.80 (22.41)	29.11 (23.76)
MAP‐DB‐IT noncompliance	22.67 (12.71)	21.1 (13.55)	19.75 (12.14)
MAP‐DB‐IT aggression	8.85 (11.17)	7.85 (9.43)	8.49 (11.08)
MAP‐DB‐IT aggression with siblings	9.15 (11.34)	8.35 (9.91)	8.9 (11.03)
ITSEA externalizing	0.54 (0.25)	0.52 (0.26)	0.50 (0.28)
ITSEA internalizing	0.55 (0.27)	0.56 (0.25)	0.53 (0.23)
ITSEA dysregulated	0.61 (0.30)	0.60 (0.30)	0.58 (0.32)
ITSEA competence	0.72 (0.48)	0.60 (0.56)	0.50 (0.55)

Abbreviations: ITSEA, infant toddler social emotional assessment; MAP‐DB‐IT, multidimensional assessment profile of disruptive behavior infant/toddler.

#### Infant toddler social emotional assessment

For the purposes of validating the MAP‐DB‐IT, we compared various psychometric properties to the ITSEA (Briggs‐Gowan & Carter, 1998). The ITSEA measures social emotional behaviors and competencies for children 12 months–35 months, 30 days and has good internal consistency (*α* = 0.85–0.89), acceptable‐to‐excellent test‐retest reliability (*r* = 0.76–0.91), and strong inter‐rater reliability (*r* = 0.72–0.79). Autistic children were included in the normative sample and this measure has additionally been validated in an autistic sample (Briggs‐Gowan & Carter, 1998; First et al., 2013; Mottes, 2015). Autistic children were not excluded from the ITSEAs norming sample (First et al., 2013), the ITSEA has been used to evaluate disruptive behavior in young autistic toddlers (Mottes, 2015), and researchers have used the ITSEA to screen young children for autism (Kruizinga et al., 2014). Within the current study, mothers completed the ITSEA across all three time points. The ITSEA consists of four subdomains: externalizing, internalizing, dysregulation, and competence. The ITSEA was also scored based on the mean of the items answered within each subdomain (see Table 2) (Baxter, 2007).

#### Autism diagnostic observation schedule, second edition

Participants were assessed for autism severity and language level based on their ADOS‐2 scores (Lord et al., 2012). Autism severity was measured based on the ADOS‐2 comparison score (Esler et al., 2015; Gotham et al., 2009). For the Toddler Module, comparison scores correspond to little to no concern (1–3), mild to moderate concern (4–5), and moderate to severe concern (6–10). For the Module 1, comparison scores correspond to minimal to no evidence (1–2), low (3–4), moderate (5–7), and high (8–10). Child verbal status was dichotomized based on ratings of item A1 on the ADOS‐2 (score of 1–2 = verbal; score of 3–4 = preverbal) (Lord et al., 2012).

### Statistical methods

Following best practices in psychometric studies, we use an argumentative approach to evaluating validity evidence (American Educational Research Association, American Psychological Association, & National Council on Measurement in Education, 2014; Gagnier et al., 2021). As such, we evaluated multiple types of validity evidence, which when combined would support the use of the MAP‐DB‐IT among this population and context.

We first examined the internal consistency reliability of the MAP‐DB‐IT domain scores using Cronbach's alpha for each of the four subdomains: temper loss, noncompliance, aggression, and aggression with sibling items. Participant's MAP‐DB‐IT domain scores were randomly selected from one of three time points (i.e., T0, T1, or T2). Randomly selected MAP‐DB‐IT time points were chosen to ensure that Cronbach's alpha was not confounded by assessment occasion and represented the full range of scores on the scale. Table S1 provides internal consistency estimates by each time point.

To evaluate convergent validity of the MAP‐DB‐IT, with the ITSEA, we correlated scores from the T0 (i.e., baseline) time point. We then correlated the MAP‐DB‐IT and ITSEA subdomains. We also examined the correlation between the change scores for the MAP‐DB‐IT and ITSEA over time to index responsiveness via convergent change. The responsiveness via convergent change allows us to evaluate the validity of a change score. Therefore, change between the MAP‐DB‐IT and ITSEA was examined from (a) T0 to T1, and (b) T1 to T2. Based on the COSMIN guidelines for validity studies (Gagnier et al., 2021), a priori hypotheses are needed to establish convergent and responsive validity. We set an a priori hypothesis of correlations within the range of approximately 0.6 with a null offset of 0.4 (i.e., correlations with confidence intervals that contain values at or below 0.4 will not be considered supporting the validity of the MAP‐DB‐IT even if statistically significant). Finally, we calculated the magnitude of change across time for each of the MAP‐DB‐IT and ITSEA subdomains.

To assess if the convergent validity of the MAP‐DB‐IT varies by child characteristics such as severity and language level as measured by the ADOS‐2, we conducted moderation analyses using Bayesian regression models for the temper loss, noncompliance, and aggression MAP‐DB‐IT subdomains. Within our models, we regressed ITSEA externalizing domain raw score on MAP‐DB‐IT domain score, ADOS‐2 language level or the ADOS‐2 severity level (i.e., the potential moderator factors), and their interaction. We used the default priors within the R brms package which are flat, uninformative priors for each of our regression parameters and student‐*t* priors for the intercept and residual variance sigma (Bürkner, 2017). For each model, we used three chains with 100 burn in iterations and 3000 samples per chain from samples per chain from the posterior distribution of regression coefficients. Each model's convergence was evaluated via Rhat values, effective sample size values for each parameter, and trace plots. Finally, each model was evaluated for accurate data prediction via posterior predictive check plots. For all our Bayesian regressions, baseline data collected at the T0 time point were used.

## RESULTS

### Evidence supporting the internal consistency of the MAP‐DB‐IT

The most basic evidence for the reliability and validity of a measure within a new population is evidence supporting its internal structure and internal consistency. As such, we calculated Cronbach's alpha for each of the MAP‐DB‐IT subdomains. The temper loss subdomain yielded a Cronbach's alpha of *α* = 0.95 [CI: 0.94, 0.97], the non‐compliance subdomain was *α* = 0.90 [CI: 0.87, 0.93], the generic aggression subdomain was *α* = 0.93 [CI: 0.91, 0.94], and the aggression with sibling items (i.e., all 25 aggression items and an additional five aggression with siblings items) was *α* = 0.95 [CI: 0.93, 0.96].

### Convergent validity between the MAP‐DB‐IT and ITSEA

Next, we evaluated the convergent validity between the MAP‐DB‐IT and the ITSEA domain scores. Consistent with our hypotheses and expectations, we found that there was a positive correlation between the (a) ITSEA externalizing and MAP‐DB‐IT aggression domain (*r* = 0.62, *p* = 0.00 [CI: 0.47, 0.74]), (b) ITSEA externalizing and MAP‐DB‐IT aggression with siblings domain (*r* = 0.59, *p* = 0.00 [CI: 0.43, 0.72]), and (c) ITSEA dysregulation and MAP‐DB‐IT temper loss domain (*r* = 0.55, *p* = 0.00 [CI: 0.41, 0.67]). See Table 3 for full correlation matrix. Additionally, see Table 4 for the magnitude of change across time for each of the MAP‐DB‐IT and ITSEA subdomains. Next, we evaluated if the convergent validity of the MAP‐DB‐IT varied by child characteristics (i.e., ADOS‐2 language level and severity score). We conducted Bayesian regression moderation analyses where the relationship between MAP‐DB‐IT subdomains and the ITSEA externalizing domain were allowed to vary by child characteristics. None of the models, were significant, and the magnitude of the interaction effects were quite small, suggesting that the convergent validity of the MAP‐DB‐IT does not vary by child characteristics. See Table 5 for these results.

**TABLE 3:** Convergent validity and convergent change validity correlations between ITSEA and MAP‐DB‐IT subdomains.

	ITSEA externalizing	ITSEA internalizing	ITSEA dysregulated	ITSEA competence
Convergent validity				
MAP‐DB‐IT temper loss	0.43 [CI: 0.23, 0.59]	0.46 [CI: 0.27, 0.62]	0.55 [CI: 0.41, 0.67]	−0.25 [CI: −0.44, −0.14]
MAP‐DB‐IT noncompliance	0.31 [CI: 0.09, 0.49]	0.35 [CI: 0.14, 0.53]	0.37 [CI: 0.17, 0.54]	−0.10 [CI: −0.31, 0.12]
MAP‐DB‐IT aggression	0.62 [CI: 0.47, 0.74]	0.33 [CI: 0.12, 0.51]	0.49 [CI: 0.31, 0.64]	−0.16 [CI: −0.36, 0.06]
MAP‐DB‐IT aggression with sibling items	0.59 [CI: 0.43, 0.72]	0.31 [CI: 0.10, 0.49]	0.47 [CI: 0.28, 0.63]	−0.14 [CI: −0.34, 0.08]
Responsiveness/convergent change validity (T0–T1)				
MAP‐DB‐IT temper loss	0.13 [CI: −0.13, 0.36]	−0.05 [CI: −0.30, 0.20]	0.19 [CI: −0.06, 0.42]	−0.12 [CI: −0.36, 0.13]
MAP‐DB‐IT noncompliance	0.11 [CI: −0.14, 0.35]	0.01 [CI: −0.24, 0.25]	0.05 [CI: −0.20, 0.30]	−0.07 [CI: −0.31, 0.18]
MAP‐DB‐IT aggression	0.34 [CI: 0.11, 0.55]	0.04 [CI: −0.21, 0.29]	0.12 [CI: −0.13, 0.36]	−0.02 [CI: −0.26, 0.23]
MAP‐DB‐IT aggression with sibling items	0.39 [CI: 0.16, 0.58]	0.05 [CI: −0.20, 0.30]	0.16 [CI: −0.09, 0.39]	−0.09 [CI: −0.33, 0.16]
Responsiveness/convergent change validity (T1–T2)				
MAP‐DB‐IT temper loss	0.43 [CI: 0.18, 0.62]	0.34 [CI: 0.08, 0.56]	0.46 [CI: 0.22, 0.65]	0.00 [CI: −0.26, 0.27]
MAP‐DB‐IT noncompliance	0.27 [CI: 0.00, 0.50]	0.17 [CI: −0.10, 0.42]	0.27 [CI: 0.01, 0.50]	−0.20 [CI: −0.44, 0.07]
MAP‐DB‐IT aggression	0.45 [CI: 0.21, 0.64]	0.32 [CI: 0.06, 0.54]	0.31 [CI: 0.05, 0.54]	−0.02 [CI: −0.28, 0.25]
MAP‐DB‐IT aggression with sibling items	0.45 [CI: 0.21, 0.64]	0.39 [CI: 0.14, 0.60]	0.38 [CI: 0.12, 0.59]	−0.05 [CI: −0.31, 0.22]

Abbreviations: ITSEA, infant toddler social emotional assessment; MAP‐DB‐IT, multidimensional assessment profile of disruptive behavior infant/toddler.

**TABLE 4:** Means, standard deviations, and effect sizes for the MAP‐DB‐IT and ITSEA raw change scores.

	Change score T1–T0	Cohen's *d*	Change score T2–T1	Cohen's *d*
MAP‐DB‐IT temper loss	−2.56 (13.56)	−0.14	−0.31 (15.00)	−0.03
MAP‐DB‐IT noncompliance	−1.44 (10.21)	−0.13	−0.15 (11.39)	−0.4
MAP‐DB‐IT aggression	−0.78 (8.33)	−0.11	0.84 (8.51)	0.11
MAP‐DB‐IT aggression with sibling items	−0.62 (8.09)	−0.10	0.65 (8.30)	0.11
ITSEA externalizing	−0.003 (0.19)	−0.04	0.01 (0.18)	−0.02
ITSEA internalizing	0.03 (0.19)	−0.006	−0.02 (0.16)	−0.15
ITSEA dysregulated	0.04 (0.18)	0.04	−0.01 (0.15)	−0.03
ITSEA competence	0.07 (0.20)	0.26	−0.01 (0.21)	0.03

Abbreviations: ITSEA, infant toddler social emotional assessment; MAP‐DB‐IT, multidimensional assessment profile of disruptive behavior infant/toddler.

**TABLE 5:** Bayesian regression moderation models of ITSEA externalizing domain as regressed on MAP‐DB‐IT subdomains, including the ADOS‐2 language ability, and ADOS‐2 severity level.

Variable	*B*	Lower 95% CI	Upper 95% CI	R‐hat	Bulk effective sample size	Tail effective sample size
Aggression and language level						
Intercept	0.43	0.36	0.51	1.00	5892	5534
MAP‐DB‐IT aggression	0.01	0.01	0.02	1.00	6415	6345
ADOS‐2 language ability	−0.03	−0.13	0.08	1.00	5179	4972
MAP‐DB‐IT aggression * ADOS‐2 language ability	0.00	0.00	0.01	1.00	5551	5975
*R* ^2^ = 0.40, CI [0.26, 0.51]						
Aggression and ASD severity						
Intercept	0.57	0.29	0.84	1.00	3774	4149
MAP‐DB‐IT aggression	0.01	−0.01	0.03	1.00	3868	4487
ADOS‐2 severity level	−0.02	−0.05	0.02	1.00	3789	3823
MAP‐DB‐IT aggression * ADOS‐2 severity level	0.00	−0.00	0.00	1.00	3935	4761
*R* ^2^ = 0.40, CI [0.26, 0.51]						
Temper loss and language level						
Intercept	0.38	0.25	0.51	1.00	4630	5211
MAP‐DB‐IT temper loss	0.00	0.00	0.01	1.00	5094	5584
ADOS‐2 language ability	0.02	−0.15	0.20	1.00	4258	4575
MAP‐DB‐IT temper loss * ADOS‐2 language ability	0.00	−0.00	0.00	1.00	4451	5091
*R* ^2^ = 0.2, CI [0.08, 0.33]						
Temper loss and ASD severity						
Intercept	0.47	−0.05	0.99	1.00	3437	3873
MAP‐DB‐IT temper loss	0.01	−0.01	0.02	1.00	3548	4042
ADOS‐2 severity level	−0.01	−0.07	0.05	1.00	3443	3880
MAP‐DB‐IT temper loss * ADOS‐2 severity level	−0.00	−0.00	0.00	1.00	3576	4241
*R* ^2^ = 0.22, CI [0.08, 0.34]						
Noncompliance and language level						
Intercept	0.41	0.25	0.57	1.00	4757	5464
MAP‐DB‐IT noncompliance	0.00	−0.00	0.01	1.00	4918	5148
ADOS‐2 language ability	0.00	−0.20	0.21	1.00	4344	4140
MAP‐DB‐IT noncompliance * ADOS‐2 language ability	0.00	−0.01	0.01	1.00	4425	4514
*R* ^2^ = 0.12, CI [0.01, 0.23]						
Noncompliance and ASD severity						
Intercept	0.45	−0.17	1.10	1.00	3291	3654
MAP‐DB‐IT noncompliance	0.01	−0.01	0.03	1.00	3216	3556
ADOS‐2 severity level	−0.00	−0.08	0.07	1.00	3287	3794
MAP‐DB‐IT noncompliance * ADOS‐2 severity level	−0.00	0.00	0.00	1.00	3250	3791
*R* ^2^ = 0.13, CI [0.03, 0.25]						

Abbreviations: ITSEA, infant toddler social emotional assessment; MAP‐DB‐IT, multidimensional assessment profile of disruptive behavior infant/toddler.

### Responsiveness of the MAP‐DB‐IT

We evaluated the convergent change between the MAP‐DB‐IT and the ITSEA as evidence for the responsiveness of the MAP‐DB‐IT to change over time. We evaluated convergent change by correlating the subdomains from (a) T0 before intervention to T1 immediately after intervention, and (b) T1 immediately after intervention to T2 3 months after intervention. When evaluating convergent change before and after intervention (T0–T1 change), there were no correlations between the ITSEA and MAP‐DB‐IT subdomains that were greater than *r* = 0.6 (Table 3). When evaluating convergent change validity immediately after intervention and 3 months after intervention (T1–T2 change), there were no correlations between the ITSEA and MAP‐DB‐IT subdomains that were greater than *r* = 0.6 (Table 3). Some T1–T2 change correlations were within the 0.6 range such as the ITSEA externalizing subdomain and the MAP‐DB‐IT (a) temper loss subdomain (*r* = 0.43, *p* = 0.001 [CI: 0.18, 0.62]), (b) aggression subdomain (*r* = 0.45, *p* = 0.001 [CI: 0.21, 0.64]), (c) aggression with sibling items (*r* = 0.45, *p* = 0.001 [CI: 0.21, 0.64]), and (d) the temper loss subdomain (*r* = 0.46, *p* = 0.001 [CI: 0.22, 0.65]). However, across all four of these positive correlations, we fail to reject our null hypothesis insofar as the 95% confidence interval also includes the null offset value of 0.4. Therefore, the MAP‐DB‐IT subdomains are measuring change in disruptive behavior differently as compared to the ITSEA subdomains.

## DISCUSSION

The current study evaluated the validity evidence for the MAP‐DB‐IT as a dimensional approach measurement for evaluating disruptive behavior in autistic toddlers. We found excellent internal consistency across all three subdomains, suggesting the MAP‐DB‐IT measures subdomains of disruptive behavior reliably. We saw that there were positive correlations between the (a) ITSEA externalizing and MAP‐DB‐IT aggression domain, (b) ITSEA externalizing and MAP‐DB‐IT aggression with siblings domain, and (c) ITSEA dysregulation and MAP‐DB‐IT temper loss domain. Taken together, these findings suggest that the psychometric properties of the MAP‐DB‐IT have sufficient validity evidence to use as a measurement tool for autistic children with disruptive behaviors.

However, our findings suggest that the responsiveness of the MAP‐DB‐IT is different from the responsiveness of the ITSEA; they evaluate change over time differently, reducing evidence for the responsiveness of the measure. It may be that the social communication interventions had an effect on children's disruptive behavior as conceptualized by only one of the measures but not both measures. Further research should consider the evaluation of additional child outcomes outside of intervention targets (Curtis et al., 2019). Therefore, the two measurement tools may be evaluating disruptive behavior differently across time due to the effects of the social communication intervention. Alternatively, it may be that the more nuanced quantification of disruptive behavior by the MAP‐DB‐IT can assess smaller changes than the more global assessment provided by the generic externalizing behavior subdomain on the ITSEA. Future research may consider how the specific dimensional or categorical characteristics of measurement tools affect change validity. Additionally, we did not find evidence that convergent validity of the MAP‐DB‐IT varied by child characteristics (i.e., ADOS‐2 language level and severity score); suggesting that there were not significant differences between the ITSEA externalizing domain and MAP‐DB‐IT subdomain scores depending on subgroup membership for the child.

The MAP‐DB‐IT was originally created for young children within the general population ages 12–18 months (Krogh‐Jespersen et al., 2021). It is critical to evaluate measurement tools originally created for the general population as they may not generalize to subpopulations' individual characteristics or psychopathology. Therefore, our analyses aimed to evaluate the psychometric properties of the MAP‐DB‐IT within a specific clinical subgroup (e.g., autistic toddlers). As such, our study's findings have broad implications for clinicians working with young autistic children who display disruptive behaviors as they can (a) use the MAP‐DB‐IT to discriminate between early developmentally appropriate tantrums from clinically salient dysregulation, and (b) compare children's MAP‐DB‐IT scores (i.e., contrast scores of autistic children to children in the general population).

Limitations to the current study include a small sample size, which restricted our analyses to evaluating cross‐sectional convergent validity instead of item level factor analyses between the MAP‐DB‐IT and the ITSEA. Additionally, the small sample may have underpowered our moderation analyses and explain the lack of significant findings from regressing MAP‐DB‐IT subdomain on parent's concern about their child's disruptive behavior as moderated by child characteristics. Furthermore, we see a small *R* ^2^ in our MAP‐DB‐IT temper loss and noncompliance Bayesian moderation models which may suggest that each of these models do a poor job of explaining the variance in our dependent variables. Previous research has found an association between child characteristics (i.e., ADOS‐2 severity and language level) and child disruptive behavior; as such, more research investigating this association is needed (Chow & Wehby, 2019; Matson et al., 2008). Additionally, our sample was predominately male (consistent with the sex distribution of autism in the population) and White (a common limitation in research samples). However, we had a wide range of language skills, cognitive abilities, and ADOS severity scores. Taken together, our sample lacks some aspects of diversity. Future longitudinal research should consider how the MAP‐DB‐IT evaluates disruptive behavior over time for a large, diverse group of autistic children (e.g., race, ethnicity, biological sex, SES). Finally, without additional diagnostic information we were unable to evaluate the diagnostic validity of the MAP‐DB‐IT. As such, future research should consider how the MAP‐DB‐IT predicts children's additional disruptive behavior disorder diagnoses. Despite these limitations, our findings suggest the MAP‐DB‐IT is a valid dimensional approach disruptive behavior measurement tool for autistic children.

Disruptive behavior is common in autistic toddlers, however clinically significant levels of disruptive behavior can lead to negative long‐term outcomes (Ashburner et al., 2008; Chow & Wehby, 2019; Kaat & Bearss, 2020). Therefore, it is important clinicians use behavioral measurement tools with autistic toddlers to refer them to further behavioral services. Dimensional approaches, such as the MAP‐DB‐IT, provide a developmentally informed metric for characterizing disruptive behavior on a spectrum from developmentally expected typical behavior to unexpected (or atypical) clinically significant concerns. However, there are few dimensional approaches validated in a young autistic sample. The current study provides preliminary psychometric validation to extend the use of the MAP‐DB‐IT to autistic toddlers. As such, the MAP‐DB‐IT may be used by a variety of clinicians as a measurement tool for autistic toddlers who may benefit from additional behavioral services or further clinical evaluations.

## AUTHOR CONTRIBUTIONS

**Hannah Fipp‐Rosenfield**: Conceptualization; data curation; formal analysis; writing – original draft. **Jeffrey Grauzer**: Data curation; formal analysis; methodology; software; writing – review & editing. **Megan Y. Roberts**: Conceptualization; funding acquisition; investigation; project administration; resources; supervision; writing – review & editing. **Aaron J. Kaat**: Conceptualization; formal analysis; methodology; supervision; writing – review & editing.

## CONFLICT OF INTEREST STATEMENT

The authors have declared they have no competing or potential conflicts of interest.

## ETHICAL CONSIDERATIONS

Ethical approval for this study was obtained from the Institutional Review Board (IRB) of Northwestern University. All participants provided informed consent prior to their participation, and their confidentiality and anonymity were strictly maintained throughout the research process.

## Supporting information

Table S1

## Data Availability

As per funding requirements, all study data are deposited in the NIMH Data Archive Collection #2335: https://nda.nih.gov/edit_collection.html?=2335.
