# Supplementary material for: Validity of the multidimensional assessment profile of disruptive behavior in autistic toddlers
Source: JCPP Adv. 2024 Apr 15;4(2):e12233. doi: 10.1002/jcv2.12233 (PMC11143949; doi:10.1002/jcv2.12233)
Supplement: Supplementary file 1 — Table S1 [file JCV2-4-e12233-s001.docx]

**Supplemental Table 1.** Internal Consistency of MAP-DB-IT Subdomains Across All Time Points

|  | **T0** | **T1** | **T2** |
| --- | --- | --- | --- |
| MAP-DB-IT Temper Loss | 0.95  [CI: 0.94, 0.97] | 0.96  [CI: 0.95, 0.97] | 0.97  [CI: 0.95, 0.98] |
| MAP-DB-IT Noncompliance | 0.90  [CI: 0.86, 0.93] | 0.92  [CI: 0.89, 0.94] | 0.91  [CI: 0.87, 0.94] |
| MAP-DB-IT Aggression | 0.93  [CI: 0.90, 0.93] | 0.92  [CI: 0.89, 0.94] | 0.92  [CI: 0.88, 0.94] |
| MAP-DB-IT Aggression with sibling items | 0.95  [CI: 0.92, 0.96] | 0.94  [CI: 0.91, 0.96] | 0.94  [CI: 0.91, 0.96] |
